# Correlation between serum cyclophilin A and acute ST-segment elevation myocardial infarction and its predictive value

**DOI:** 10.3389/fcvm.2025.1566129

**Published:** 2025-05-27

**Authors:** Yuqi Wang, Long Wang, Caoyang Fang, Zhenfei Chen

**Affiliations:** ^1^ Graduate School, Bengbu Medical University, Bengbu, Anhui, China; ^2^ Department of Cardiology, The Second People’s Hospital of Hefei, Hefei Hospital Affiliated to Anhui Medical University, Hefei, Anhui, China; ^3^ Department of Emergency, The First Affiliated Hospital of USTC, Division of Life Sciences and Medicine, University of Science and Technology of China, Hefei, Anhui, China

**Keywords:** CyPA, cyclophilin A, STEMI, ST-segment elevation myocardial infarction, prediction

## Abstract

**Objective:**

Acute ST-segment elevation myocardial infarction (STEMI) is a serious cardiovascular event, and timely diagnosis and intervention are essential to improve prognosis. As a novel biomarker, serum cyclophilin A (CyPA) may play an important role in the development and progression of STEMI.

**Methods:**

Two hundred and sixty-four patients with acute STEMI and 264 healthy controls were included in this study. The association of CyPA with STEMI was assessed by multivariate logistic regression analysis, and the potential value of CyPA in predicting STEMI risk was assessed using receiver operating characteristic curve (ROC) and reclassification analysis. In addition, calibration curves and decision curve analysis (DCA) were used to test the reliability of CyPA in predicting STEMI risk.

**Results:**

The results showed that serum CyPA levels were significantly higher in patients with acute STEMI than in healthy controls (*P* < 0.001). Multivariate logistic regression analysis indicated that CyPA level was an independent risk factor for acute STEMI (OR = 1.15, 95% CI: 1.09, 1.23). In Model 3, each unit increase in CyPA was associated with a 17% increased risk of STEMI (*P* < 0.0001). Compared with Q1, the Q4 group (OR = 157.45, 95% CI: 65.19, 420.54) had a dramatically increased risk of STEMI. CyPA was found to have a non-linear relationship throughout the range by restricted cubic spline (RCS) analysis. The AUC of ROC curve analysis in model C was 0.994, which was significantly improved compared with model B (*P* = 0.01); the net reclassification index (NRI) was 0.0682 (*P* = 0.00029), and the comprehensive discriminant improvement index (IDI) was 0.0438 (*P* < 0.0001). The calibration curve showed that model C was more stable, and DCA showed that model C had better net yield, which was superior to model A and model B.

**Conclusion:**

This study showed that serum cyclophilin A levels were closely associated with the development of acute ST-segment elevation myocardial infarction. CyPA may become a potential biomarker for acute STEMI.

## 1 Introduction

Serum cyclophilin A (CyPA), an important cytokine, has attracted significant attention in cardiovascular disease research in recent years. Acute ST-segment elevation myocardial infarction (STEMI) is a serious cardiovascular event characterized by a complex pathogenesis involving various physiological and pathological processes. CyPA plays a critical role in myocardial ischemia and reperfusion injury, and elevated levels of CyPA in STEMI patients suggest its potential as a biomarker (1, 2). Research indicates that CyPA is not only involved in inflammatory responses but is also closely related to processes such as cell apoptosis and cardiac remodeling, which may position it as a key player in the occurrence and progression of STEMI (3).

Clinical studies have shown that serum levels of CyPA are closely associated with the occurrence, severity, and prognosis of STEMI. One study indicated that CyPA levels in STEMI patients were significantly higher than those in healthy controls and positively correlated with the severity of myocardial injury (4). Moreover, changes in CyPA levels in patients with acute coronary syndrome (ACS) have been regarded as important indicators for assessing disease stability and prognosis, further emphasizing its significance in clinical applications (5). Elevated levels of CyPA are not only related to the severity of myocardial injury but may also serve as an independent risk factor for predicting cardiovascular events (6, 7). For instance, some studies have found that increased CyPA levels are associated with worsening left ventricular function after myocardial infarction, suggesting its potential role in the cardiac remodeling process (8, 9).

The clinical application of CyPA holds great promise, particularly in the early identification of high-risk patients and optimization of treatment strategies. By monitoring CyPA levels, clinicians can better assess the risk of STEMI patients, enabling the development of personalized treatment plans. The use of this biomarker not only aids in improving the early diagnosis of STEMI but may also enhance patient outcomes and reduce the incidence of cardiovascular events (10, 11). Although some studies have shown that CyPA has certain correlation in cardiovascular diseases such as coronary heart disease, the clinical application value of CyPA has not been systematically verified in the field of early diagnosis and risk prediction of acute ST-segment elevation myocardial infarction (STEMI). Most previous studies have focused on disease-related analysis, lacking a large sample size, and a comprehensive assessment of the independent efficacy and incremental value of CyPA in STEMI risk stratification by multidimensional statistical tools. In addition, the clinical discrimination and stability of CyPA combined with traditional risk factors are rarely quantitatively analyzed by NRI, IDI and decision curve at home and abroad.Therefore, a deeper investigation into the mechanisms by which CyPA functions in STEMI and its potential as a biomarker is of significant clinical importance and necessity for advancing early diagnosis and intervention in cardiovascular diseases. Consequently, this study aims to explore the correlation between CyPA and acute ST-segment elevation myocardial infarction, as well as its value in risk prediction.

## 2 Study subjects and methods

### 2.1 Study subjects

This study conducted a retrospective analysis of 264 STEMI patients who underwent emergency percutaneous coronary intervention (PCI) at our hospital between December 2021 and December 2024, serving as the experimental group. The inclusion criteria were as follows: (1) All patients met the diagnostic criteria for ST-segment elevation myocardial infarction (STEMI) in the Practice for the Diagnosis and Treatment of Acute Myocardial Infarction (12). Specifically, they presented with typical clinical symptoms of myocardial ischemia (e.g., chest pain), elevated markers of myocardial injury (e.g., troponin), and ST segment elevation in at least two leads on the electrocardiogram. In addition, all included patients received standardized treatment in strict accordance with the above guidelines, including timely reperfusion therapy [preferred percutaneous coronary intervention (PCI) or thrombolytic therapy if necessary] and secondary preventive measures, such as antiplatelet drugs, statins, β-blockers, and angiotensin converting enzyme inhibitors or receptor antagonists (ACEI/ARBs); (2) patients completed myocardial enzyme profiles, troponin tests, coronary angiography, and electrocardiography, and were able to tolerate emergency PCI; (3) patients were aged 18 years or older; and (4) patients' clinical data were complete. Exclusion criteria included: (1) a history of myocardial infarction; (2) severe metabolic disorders; (3) significant organ dysfunction, such as severe liver or kidney impairment; (4) severe infections; (5) coagulation disorders; and (6) recent history of major surgery or trauma. Additionally, we selected a control group consisting of 284 patients who presented with chest pain during the same period and had negative results from coronary angiography or coronary CT angiography. To ensure the rigor of the study, a retrospective calculation was performed using the logistic regression binary variable sample size formula based on a two-sided alpha of 0.05, a power of 80%, and an expected OR of 1.7 (case-control ratio 1:1) for CyPA and STEMI suggested in the literature, resulting in a minimum required sample size of 206 (103 in each group). The actual sample size in this study far exceeds the minimum requirement, ensuring the reliability and statistical power of the analysis. This study was approved by the Ethics Committee of the Second People's Hospital of Hefei (2020-KE-058). Informed consent was obtained from all participants and/or their legal guardians. Studies involving human research participants were conducted in accordance with the Declaration of Helsinki.

### 2.2 Study methods

#### 2.2.1 Data collection

Through the hospital's electronic medical record system, we collected the basic demographic characteristics and clinical information of STEMI patients at the time of admission. This information included the patients' age, gender, body mass index (BMI), smoking and drinking habits, as well as histories of hypertension and diabetes. Additionally, we recorded various hematological parameters, such as neutrophils, lymphocytes, monocytes, hemoglobin, and platelets. Furthermore, we included data on glycated hemoglobin, albumin, urea, creatinine, uric acid, total and direct bilirubin, as well as triglycerides (TG), total cholesterol (TC), high-density lipoprotein (HDL), low-density lipoprotein (LDL), estimated glomerular filtration rate (eGFR), left ventricular ejection fraction (LVEF), fasting plasma glucose (FPG), glycated hemoglobin (HbA1c), and cystatin C (CysC).

#### 2.2.2 Serum cyclophilin A (CyPA) determination

The CyPA level was measured using the enzyme-linked immunosorbent assay (ELISA) method, with kits provided by CUSABIO. The procedure was strictly followed as outlined below:

(1.) Allow all reagents to reach room temperature (18–25°C) for at least 30 min, and prepare the reagents as previously described. (2.) Sample addition: Set up wells for the standard and test samples. Add 100 µl of either the standard or test sample to each well, gently mix by shaking, then cover with a plate seal and incubate at 37°C for 2 h. (3.) Dispose of the liquid and dry the wells without washing. (4.) Add 100 µl of biotin-labeled antibody working solution to each well, cover with a new plate seal, and incubate at 37°C for 1 h. (5.) Discard the liquid from each well, dry them, and wash the plate 3 times, with each wash soaking for 2 min at 200 µl per well, then dry again. (6.) Add 100 µl of horseradish peroxidase-labeled streptavidin working solution to each well, cover with a new plate seal, and incubate at 37°C for 1 h. (7.) Remove the liquid from each well, dry them, and wash the plate 5 times, each wash soaking for 2 min at 200 µl per well, then dry again. (8.) Sequentially add 90 µl of substrate solution to each well and allow color development at 37°C in the dark for 15–30 min. (9.) Sequentially add 50 µl of stop solution to each well to terminate the reaction. (10.) Within 5 min of stopping the reaction, measure the optical density (OD value) of each well at a wavelength of 450 nm using a microplate reader. Finally, use the OD values of the samples to determine their corresponding concentrations from the standard curve, or calculate the regression equation for the standard curve using the concentration of the standards and their OD values, and then substitute the sample OD values into the equation to obtain the sample concentrations.

#### 2.2.3 Models establishment and validation

Multivariate logistic regression analysis was used to identify independent risk factors for STEMI and to establish three predictive models: Model A is the CyPA model, Model B is based on independent clinical risk factors, and Model C combines CyPA with clinical risk factors. The clinical applicability of Model C was assessed using the net reclassification improvement index (NRI), integrated discrimination improvement index (IDI), receiver operating characteristic curve (ROC), calibration curve, and decision curve analysis (DCA). An NRI value greater than 0 indicates that the new model is superior to the old model, while a negative value suggests the opposite. The IDI reflects the change in the predictive ability difference between the two models; a higher value indicates a stronger predictive capability of the new model. Specifically, an IDI greater than 0 signifies an improvement, less than 0 indicates a negative improvement, and equal to 0 suggests no change. For details regarding the clinical risk factors identified through multivariate logistic regression analysis, please refer to Supplementary Table S1.

#### 2.2.4 Statistical methods

Statistical analyses and graphing were performed using SPSS 26.0 and R 4.2.1. Measurement data were expressed as mean ± standard deviation, independent sample t-test was used for comparison between the two groups; enumeration data adoption rate (%) was expressed, chi-square test was used for comparison between the two groups. Additionally, Univariate and multivariate logistic regression analyses were used to predict independent factors for STEMI risk. CyPA levels were categorized into four groups based on quartiles (Q1, Q2, Q3, Q4), with Q1 serving as the control group. The relationship between CyPA and STEMI risk was analyzed using multivariate logistic regression models: Model 1 included no adjustments for covariates; Model 2 adjusted for age, sex, BMI, smoking, drinking, hypertension, and diabetes; and Model 3 further adjusted for neutrophils, albumin, fasting plasma glucose (FPG), LDL, HDL, and left ventricular ejection fraction (LVEF). The relationship between CyPA and STEMI risk was explored across the entire range using restricted cubic splines (RCS). We also conducted receiver operating characteristic (ROC) curve analysis and reclassification analysis to assess the potential value of CyPA in predicting STEMI risk, including the net reclassification index (NRI) and integrated discrimination improvement (IDI). Reliability was assessed through calibration curves and decision curve analysis (DCA) for the prediction of STEMI risk with CyPA. All statistical analyses were two-tailed, and a *P*-value of <0.05 was considered statistically significant.

## 3 Results

### 3.1 Characteristics of study participants

This study included a total of 528 participants, with 264 in the control group and 264 in the STEMI group. The basic characteristics of the participants are as follows: the mean age of the STEMI group was 57.86 ± 13.10 years, while that of the control group was 61.83 ± 10.62 years (*P* < 0.001). Regarding gender, there were 368 males (69.70%) and 160 females (30.30%), with a significantly higher proportion of males in the STEMI group compared to the control group (*P* < 0.001). In terms of body mass index (BMI), the average BMI of the STEMI group was 25.35 ± 3.79, while the control group had an average BMI of 25.93 ± 3.51 (*P* = 0.069). Concerning hematological parameters, the STEMI group exhibited significantly higher levels of neutrophils, monocytes, and hemoglobin compared to the control group (*P* < 0.001). Among the biochemical indices, direct bilirubin, indirect bilirubin, creatinine, uric acid, and fasting blood glucose levels were all significantly higher in the STEMI group than in the control group (*P* < 0.05). In terms of lifestyle, there were 308 smokers (58.33%), 101 alcohol consumers (19.13%), 180 patients with hypertension (34.09%), and 259 patients with diabetes (49.05%) among the participants. The incidence of hypertension and diabetes was significantly higher in the STEMI group than in the control group (*P* < 0.001). Additionally, the level of CyPA in the STEMI group (22.01 ± 9.68) was significantly higher than that in the control group (13.89 ± 7.25), with *P* < 0.05. Detailed results can be found in Table 1.

**Table 1:** Comparison of general clinical characteristics between STEMI group and Non-STEMI group.

Variables	Total (*n* = 528)	Non-STEMI (*n* = 264)	STEMI (*n* = 264)	Statistic	*P*
Age, mean ± SD	59.84 ± 12.08	61.83 ± 10.62	57.86 ± 13.10	3.83	<0.001
BMI, mean ± SD	25.64 ± 3.66	25.93 ± 3.51	25.35 ± 3.79	1.82	0.069
Neutrophils, mean ± SD	6.16 ± 3.14	4.19 ± 1.80	8.13 ± 2.95	−18.53	<0.001
Lymphocytes, mean ± SD	1.74 ± 0.79	1.78 ± 0.56	1.71 ± 0.97	1.00	0.320
Monocyte, mean ± SD	0.49 ± 0.22	0.40 ± 0.17	0.59 ± 0.23	−10.57	<0.001
HB, mean ± SD	134.46 ± 16.18	131.11 ± 15.20	137.82 ± 16.45	−4.87	<0.001
Platelets, mean ± SD	203.95 ± 72.40	197.60 ± 72.18	210.31 ± 72.21	−2.02	0.044
Albumin, mean ± SD	39.93 ± 4.11	40.79 ± 4.48	39.07 ± 3.50	4.89	<0.001
Creatinine, mean ± SD	70.33 ± 18.08	67.03 ± 15.37	73.62 ± 19.93	−4.26	<0.001
BUN, mean ± SD	5.89 ± 1.95	5.74 ± 1.55	6.03 ± 2.28	−1.75	0.080
eGFR, mean ± SD	92.69 ± 17.48	91.86 ± 16.01	93.52 ± 18.84	−1.09	0.276
UA, mean ± SD	347.32 ± 107.17	336.08 ± 111.45	358.57 ± 101.70	−2.42	0.016
FPG, mean ± SD	6.43 ± 2.51	5.83 ± 1.74	7.04 ± 2.97	−5.72	<0.001
TG, mean ± SD	1.88 ± 0.98	1.80 ± 1.01	1.95 ± 0.95	−1.71	0.088
TC, mean ± SD	4.36 ± 1.11	4.12 ± 1.13	4.60 ± 1.04	−5.06	<0.001
LDL, mean ± SD	2.83 ± 0.97	2.57 ± 0.92	3.09 ± 0.94	−6.38	<0.001
HDL, mean ± SD	1.11 ± 0.27	1.19 ± 0.29	1.04 ± 0.24	6.15	<0.001
HbA1c, mean ± SD	6.67 ± 1.70	6.74 ± 1.83	6.60 ± 1.55	0.99	0.324
LVEF, mean ± SD	60.08 ± 7.56	62.66 ± 4.83	57.50 ± 8.83	8.32	<0.001
CyPA, mean ± SD	17.95 ± 9.46	13.89 ± 7.25	22.01 ± 9.68	−10.91	<0.001
Sex, *n* (%)				51.80	<0.001
Female	160 (30.30)	118 (44.70)	42 (15.91)		
Male	368 (69.70)	146 (55.30)	222 (84.09)		
Smoke, *n* (%)				1.99	0.158
No	220 (41.67)	118 (44.70)	102 (38.64)		
Yes	308 (58.33)	146 (55.30)	162 (61.36)		
Alcohol, *n* (%)				29.40	<0.001
No	427 (80.87)	189 (71.59)	238 (90.15)		
Yes	101 (19.13)	75 (28.41)	26 (9.85)		
Hypertension, *n* (%)				151.35	<0.001
No	348 (65.91)	241 (91.29)	107 (40.53)		
Yes	180 (34.09)	23 (8.71)	157 (59.47)		
Diabetes, *n* (%)				150.67	<0.001
No	269 (50.95)	64 (24.24)	205 (77.65)		
Yes	259 (49.05)	200 (75.76)	59 (22.35)		

BMI, body mass index; HB, hemoglobin; BUN, blood urea nitrogen; eGFR, estimated glomerular filtration rate; UA, uric acid, FPG, fasting plasma glucose; TG, triglyceride; TC, total cholesterol; HDL, high density lipoprotein; LDL, low density lipoprotein; HbA1c, glycosylated hemoglobin; LVEF, left ventricular ejection fraction; CyPA, serum cyclophilin A.

### 3.2 Relationship between CyPA and STEMI risk

Table 2 presents the correlation between CyPA levels and the risk of STEMI. In Model 1, for each 1-unit increase in CyPA, the risk of STEMI rises by 11% (OR: 1.11, 95% CI: 1.09–1.14). In Model 2, this risk increases by 13% (OR: 1.13, 95% CI: 1.09–1.16). In the further adjusted Model 3, for each additional unit of CyPA, the risk of STEMI increases by 17% (OR: 1.17, 95% CI: 1.14–1.22). In the quartile analysis, the highest quartile (Q4) of CyPA demonstrates a significantly increased risk of STEMI compared to the lowest quartile (Q1) (OR: 157.45, 95% CI: 65.19–420.54).

**Table 2:** Relationship between CyPA and STEMI risk.

Variables	Model 1		Model 2		Model 3	
	OR (95% CI)	*P*	OR (95% CI)	*P*	OR (95% CI)	*P*
CyPA	1.11 (1.09, 1.14)	<0.0001	1.13 (1.09, 1.16)	<0.0001	1.17 (1.14, 1.22)	<0.0001
CyPA category						
Q1	Ref	Ref	Ref	Ref	Ref	Ref
Q2	1.53 (0.89, 2.63)	0.12	1.36 (0.64, 2.91)	0.42	1.63 (0.79, 3.42)	0.19
Q3	3.32 (1.97, 5.69)	<0.0001	5.38 (2.54, 11.90)	<0.0001	12.24 (5.64, 28.14)	<0.0001
Q4	34.38 (17.07, 75.31)	<0.0001	66.23 (25.21, 192.64)	<0.0001	157.45 (65.19, 420.54)	<0.0001
*P* for trend	<0.0001		<0.0001		<0.0001	

OR, odds ratio; CI, confidence interval; Ref, reference.

Model 1: No adjustments made.

Model 2: Adjusted for age, sex, BMI, smoke, alcohol, hypertension, diabetes.

Model 3: Adjusted for age, sex, BMI, smoke, alcohol, hypertension, diabetes, neutrophils, albumin, FPG, LDL, HDL, CysC, LVEF.

### 3.3 RCS analysis of the association between CyPA and STEMI risk

**Figure 1:**
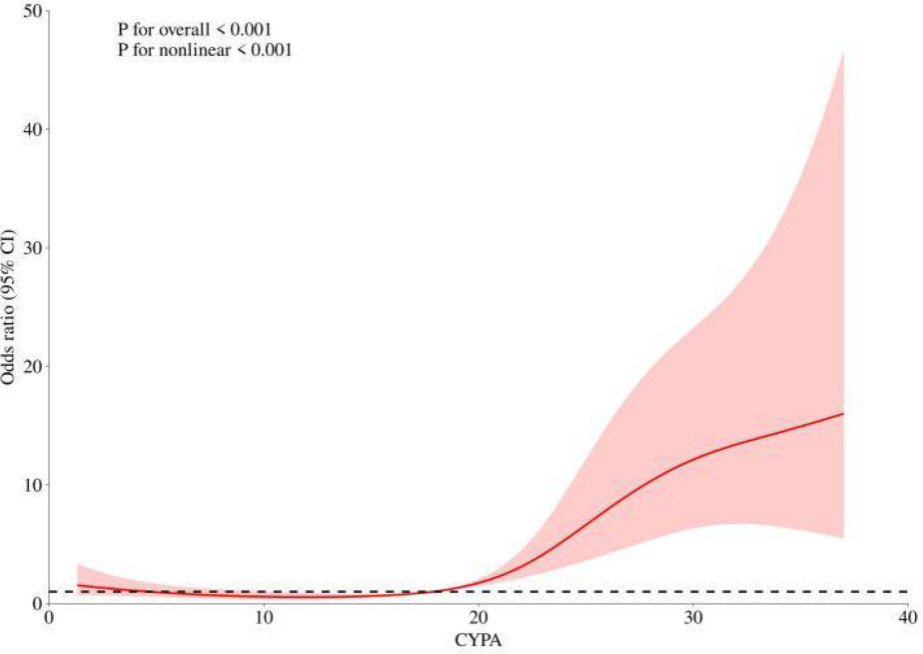
RCS analysis of the association between CyPA and STEMI risk.

According to Table 2, there is a clear increasing trend in STEMI risk from Q1 to Q4. To further validate the relationship between CyPA and STEMI risk, we performed a smooth curve fitting analysis. Figure 1 illustrates that as the CyPA values increase, the risk of STEMI shows a nonlinear upward trend. The statistical tests also confirm this nonlinear relationship (Non-linearity *P* < 0.001).

### 3.4 ROC and reclassification analyses

**Figure 2:**
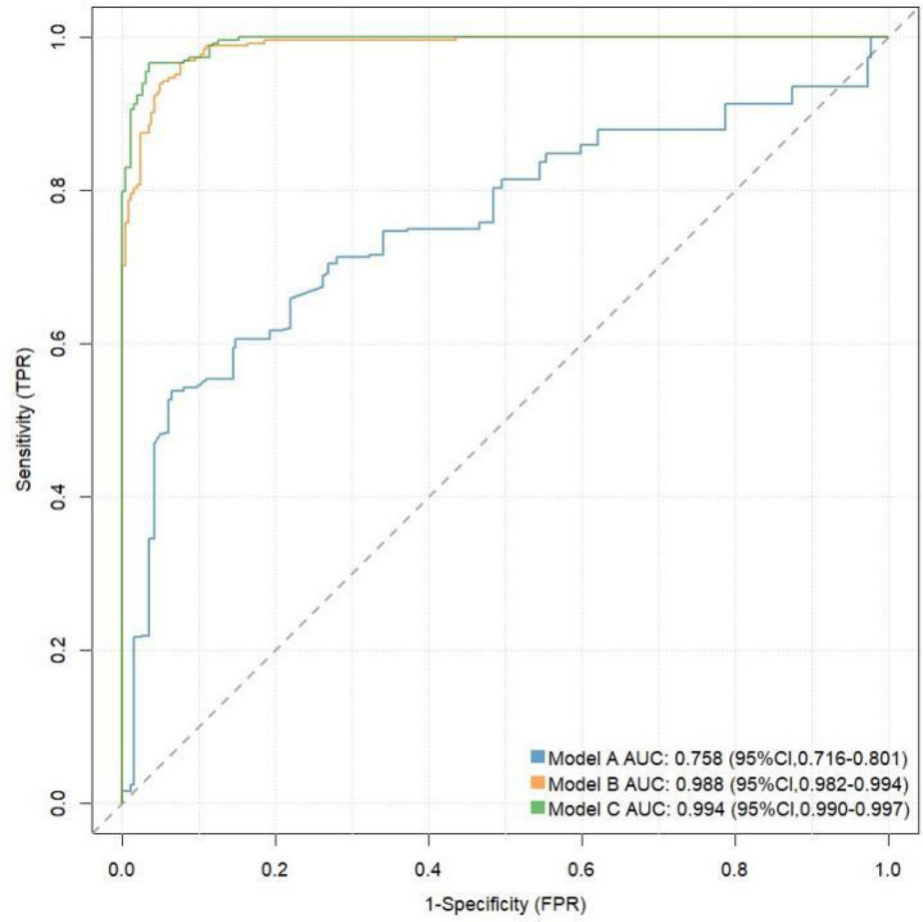
ROC curve analysis.

We assessed the value of CyPA in predicting the risk of STEMI using ROC curves and reclassification analysis (see Table 3 and Figure 2). In the ROC analysis, the area under the curve (AUC) for Model A was 0.758 (95% CI: 0.716–0.801), Sensitivity: 0.540, specificity: 0.935, Youden Index: 0.475; Cut-off Value: 0.644; while Model B had an AUC of 0.988 (95% CI: 0.982–0.994), Sensitivity:0.962, specificity:0.927,Youden Index: 0.889; Cut-off Value: 0.399; and Model C achieved an AUC of 0.994 (95% CI: 0.990–0.997),Sensitivity:0.962, specificity: 0.943, Youden Index: 0.905; Cut-off Value: 0.382;. In the reclassification analysis, the net reclassification index (NRI) was 0.0682 (95% CI: 0.0313–0.105), and the integrated discrimination improvement (IDI) was 0.0438 (95% CI: 0.0263–0.0613), further indicating that CyPA significantly improves the prediction of STEMI risk.

**Table 3:** ROC curve and reclassification analysis for three models.

Models	AUC (95% CI)	*P* value	*P* for comparision	NRI (categorical)	*P* value	IDI	*P* value
Model A	0.758 (0.716, 0.801)	<0.0001	–	–	–	–	–
Model B	0.988 (0.982, 0.994)	<0.0001	–	–	–	–	–
Model C	0.994 (0.990, 0.997)	<0.0001	0.01^#^	0.0682 (0.0313, 0.105)	0.00029	0.0438 (0.0263, 0.0613)	<0.0001

Model B, clinical risk factors; Model C, clinical risk factors + CyPA; clinical risk factors, alcohol, hypertension, diabetes, neutrophils, albumin, FPG, LDL, HDL, LVEF; NRI, net reclassification index, IDI: integrated discrimination improvement.

^#^ Indicates comparison to Model B.

### 3.5 Calibration curve and decision curve analysis

**Figure 3:**
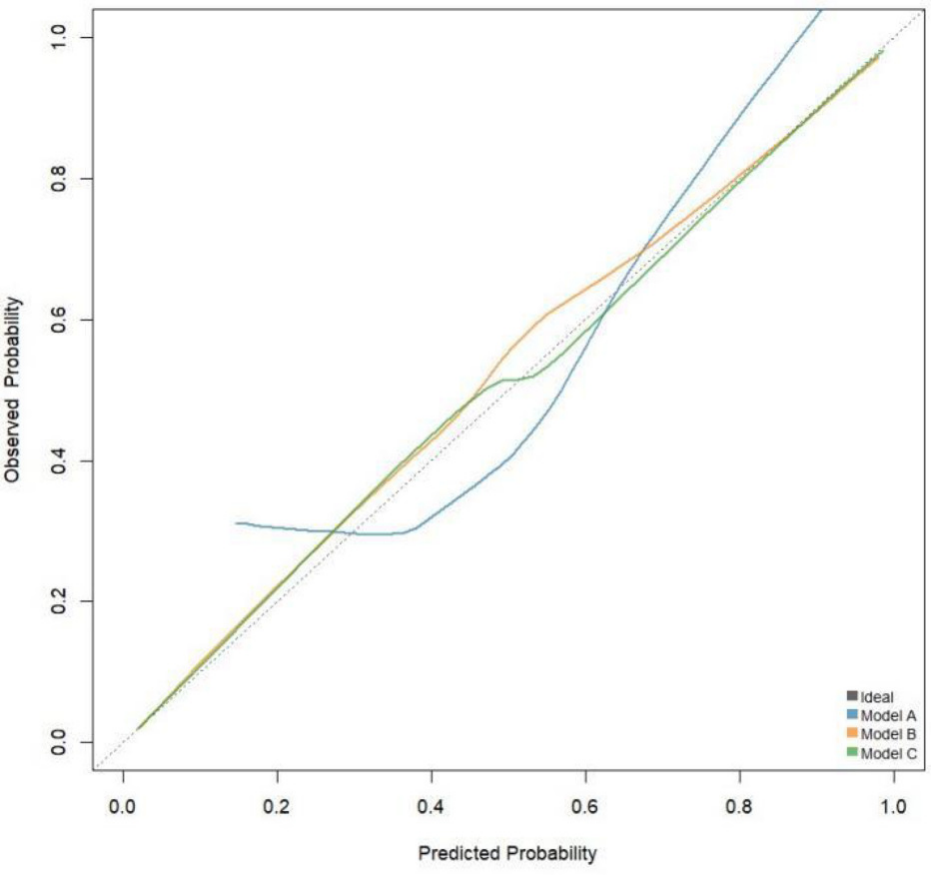
Calibration curve.

**Figure 4:**
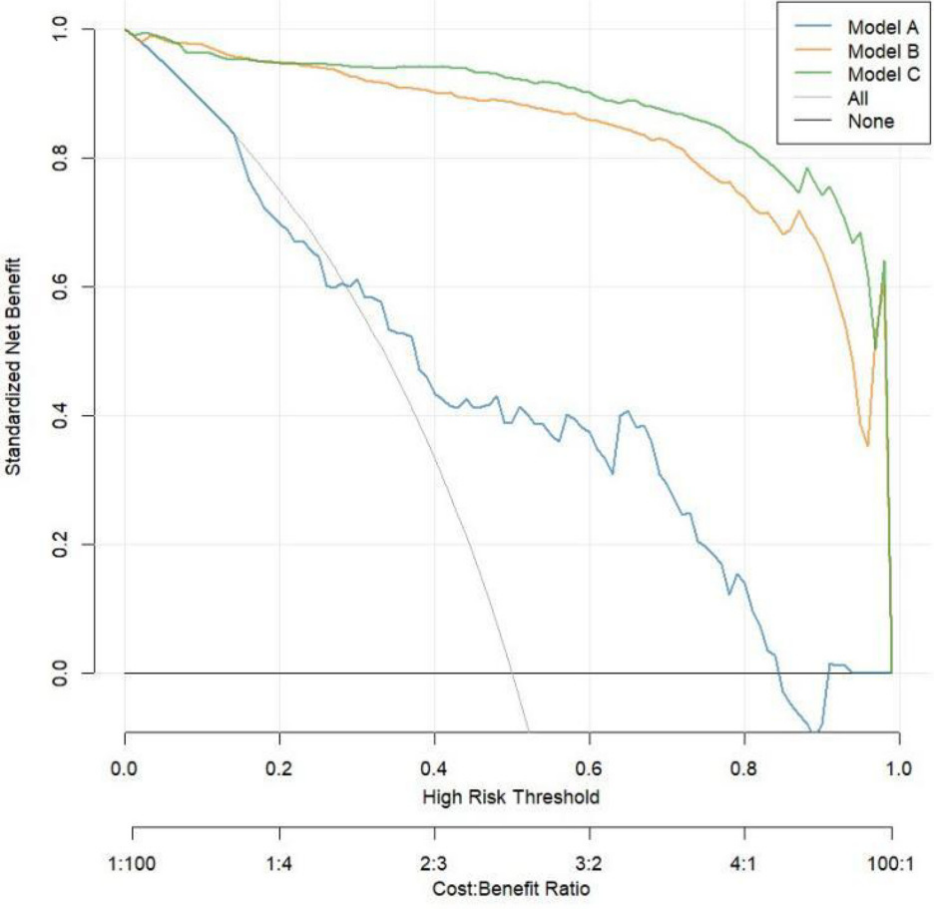
Decision curve analysis.

The calibration curve shown in Figure 3 indicates that Model C is closer to the ideal curve compared to Models A and B, suggesting that Model C has a higher stability. Figure 4 presents the decision curves for Models A, B, and C when predicting the risk of STEMI. All models demonstrate effectiveness across the threshold probability range of 1% to 99%, and within this range, Model C provides a greater net benefit than Models A and B. This indicates that when the threshold probability is set between 1% and 99%, Model C can deliver significant clinical net benefits for patients.

## 4 Discussion

This study systematically investigated the value of CyPA in predicting the risk of STEMI. The results showed that CyPA levels were significantly higher in STEMI patients than in controls; CyPA was able to predict the occurrence of STEMI as an independent risk factor, and this association remained robust even after accounting for traditional cardiovascular risk factors. Further analysis showed that the discriminatory ability and clinical application value of CyPA were significantly improved after it was included in the traditional risk prediction model. Taken together, this study supports the potential of CyPA as a novel biomarker for STEMI risk assessment and provides new ideas and evidence for precise stratification and early warning of cardiovascular disease.These findings are consistent with previous research, which also highlighted the important role of CyPA in cardiovascular diseases, particularly in the context of acute coronary syndrome and myocardial infarction (13–15).

CyPA is an important cytokine that has garnered significant attention in cardiovascular disease research in recent years. CyPA not only plays a vital biological role within cells, but also participates in various pathological processes through its extracellular actions. Studies have demonstrated that CyPA is crucial in myocardial ischemia and reperfusion injury, and its elevated levels in patients with acute ST-segment elevation myocardial infarction (STEMI) suggest its potential as a biomarker (2, 16). CyPA promotes inflammatory responses and platelet activation by interacting with receptors such as CD147 (EMMPRIN) and RAGE (receptor for advanced glycation end products), thereby playing an essential role in the onset and progression of cardiovascular diseases (3, 17, 18). Furthermore, the release of CyPA is closely associated with oxidative stress; this oxidative stress not only stimulates CyPA secretion but also exacerbates myocardial injury and inflammatory responses by enhancing its extracellular activity (19, 20). Therefore, monitoring CyPA levels may provide new insights for the early diagnosis and risk assessment of cardiovascular diseases.

Acute ST-segment elevation myocardial infarction (STEMI) is a serious cardiovascular event characterized by a complex pathogenesis involving various physiological and pathological processes. The occurrence of STEMI is closely linked to inflammatory responses, oxidative stress, and platelet activation, with CyPA playing a significant role in these processes. Research has shown that CyPA levels in STEMI patients are significantly higher than those in healthy controls and are positively correlated with the severity of myocardial injury (10, 21). In one study, elevated CyPA was identified as an independent risk factor for predicting STEMI, and its changes were considered important indicators for assessing disease stability and prognosis in patients with acute coronary syndrome (ACS) (11, 22). Furthermore, alterations in CyPA levels have shown potential clinical value in the prognostic assessment of STEMI patients, particularly concerning cardiac remodeling and functional recovery (23, 24). Therefore, in-depth investigation into the mechanisms of CyPA in STEMI and its potential as a biomarker holds significant clinical importance for advancing early diagnosis and intervention in cardiovascular diseases.

In recent years, a growing body of evidence has explored the role of Cyclophilin A (CyPA) in cardiovascular diseases, particularly in the context of acute ST-segment elevation myocardial infarction (STEMI). Several international studies, such as Satoh et al. (2013) and Shinohara et al. (2010), have reported that plasma CyPA levels are significantly elevated in patients with acute coronary syndromes compared to controls and may serve as a novel biomarker of coronary artery disease activity (5, 25). The strengths of these studies include their innovative identification of CyPA as a potential clinical marker and well-defined study endpoints. However, their sample sizes were relatively small, and most were conducted in single centers and among predominantly Caucasian populations, which may limit the generalizability of their findings. In addition, some lacked comprehensive adjustment for conventional cardiovascular risk factors or detailed assessment of CyPA's contribution to risk prediction models.

In exploring the mechanisms of action of CyPA, the study found that it interacts with receptors such as CD147 (EMMPRIN) and RAGE (receptor for advanced glycation end products) to promote inflammation and platelet activation (5, 26, 27). The release of CyPA is closely related to oxidative stress, which not only enhances the secretion of CyPA but also exacerbates myocardial injury and inflammatory responses by increasing its extracellular activity (28–31). This research validated the nonlinear relationship between CyPA and STEMI risk through smooth curve fitting, further supporting the complex mechanisms by which CyPA operates in cardiovascular diseases.

In addition, this study evaluated the predictive value of CyPA for STEMI risk using ROC curves and reclassification analysis. This indicates the potential of CyPA as a biomarker, surpassing previous research findings (2, 32). Model A with CyPA alone as a predictor had significantly weaker discriminatory power than Model B based on multiple clinical variables, a phenomenon that is in line with current consensus in the field of coronary heart disease risk prediction. Traditional clinical risk factors (e.g., age, sex, hypertension, diabetes, smoking, dyslipidemia, etc) are able to reflect the pathogenesis of STEMI from multiple levels and thus have strong independence and broad applicability for risk judgment (33). When CyPA was added further to the clinical model (Model B), even if it brought only a slight ROC improvement (Model C), it suggests that CyPA may have a gain effect on STEMI risk identification. It should be pointed out that when the basic clinical model has performed very well, it is often difficult to achieve significant improvement with the addition of a single biomarker, which is in line with a marginal decreasing effect. However, even small improvements in model performance may complement individualized decision-making in specific clinical scenarios such as screening of high-risk populations and subgroup assessment. Therefore, CyPA can be used as a supplement to existing clinical assessment tools, and large samples and multicenter cohorts are needed to further confirm its clinical value in the future. The calibration and decision curve analyses showed that model C closely aligned with the ideal curve compared to models A and B, demonstrating its stability and effectiveness in clinical applications. These results are consistent with earlier studies that also highlighted the importance of CyPA in assessing cardiovascular disease risk (34, 35).

Interestingly, our results showed higher hemoglobin levels in STEMI patients. Multiple explanations for this phenomenon may exist. First, during acute myocardial infarction, dehydration or decreased plasma volume due to stress, vomiting, or decreased food intake leads to hemoconcentration, which increases the surface of hemoglobin (36). Second, increased hemoglobin may be associated with chronic hypoxic conditions, such as smoking patients or patients with chronic lung diseases, which are also high risk factors for coronary heart disease (37, 38). In addition, large cohort studies have shown a possible J-shaped relationship between hemoglobin levels and cardiovascular risk, ie, both anemia and polycythemia are associated with adverse cardiovascular outcomes (39). Therefore, the increased hemoglobin in STEMI patients in this study may be a result of a combination of the above mechanisms. Future clinical studies are needed to clarify the prognostic significance of elevated hemoglobin in this population.

Compared with the above studies, this study has the following advantages: (1) the sample size is large and the case-control is balanced, enhancing the statistical power and representativeness; (2) on the basis of combined multivariate analysis, the discriminant power and reclassification ability of CyPA after adding to the model are systematically assessed, and the clinical net benefit is analyzed using the clinical decision curve for the first time; (3) the population involved is actually hospitalized STEMI patients in mainland China, and the results are more meaningful in local practice.

The differences in the conclusions of different current studies may come from many aspects: first, the inclusion criteria and case composition are different, some studies focus on patients with specific complications, and some are small sample single-center observations. This study includes a wider range of real-world cases and reduces the selection bias; second, there are differences in the detection methods, sample collection time points and analysis means of CyPA, which may lead to different absolute values and cut-off values; third, whether different studies adjust the traditional risk factors and whether a comprehensive prediction model is constructed may affect the evaluation intensity of CyPA independent prediction value and gain effect.

We believe that CyPA, as a molecule reflecting inflammatory response and oxidative stress injury, is able to provide unique information on the pathological process of STEMI. As a complementary biomarker, CyPA is expected to play an important role in risk stratification, prognostic assessment, etc. in STEMI or suspected STEMI patients in the future. Compared with high-sensitivity troponin (hs-cTn) commonly used in clinical practice, CyPA is not only able to assist in the quantitative assessment of acute myocardial injury, but may also compensate for the limited specificity of hs-cTn in some special populations (such as patients with chronic kidney disease or those with chronic myocardial injury). In the future, the accuracy of STEMI diagnosis, risk stratification and prognosis can be improved by combining multiple biomarkers (such as combined detection of CyPA and hs-cTn). It should be pointed out that CyPA, as an emerging marker, its specific clinical application value still needs to be further validated in larger samples, multicenter and prospective studies.

However, this study has several limitations. First, sample size constraints: Although this study included 528 participants, the sample size remains relatively small, which may affect the external validity and statistical significance of the results. Second, single-center study: Conducting this research at a single center may introduce selection bias and limit the generalizability of the findings. Multicenter studies could provide more representative results. Third, lack of long-term follow-up: This study did not include long-term follow-up of patients, making it impossible to assess the relationship between CyPA levels and long-term outcomes in STEMI patients. Fourth, potential confounding factors: Although multivariable analysis was performed, there may still be uncontrolled confounding factors, such as patients' lifestyle and comorbidities, which could influence the relationship between CyPA and STEMI. Fifth, insufficient mechanistic research: This study primarily focused on the correlation and predictive value of CyPA levels, lacking an in-depth exploration of its potential mechanisms. Future research should consider investigating the biological mechanisms of CyPA in the occurrence of STEMI. Lastly, insufficient validation of clinical application: While the results indicate that CyPA has good sensitivity and specificity for predicting STEMI risk, further validation in clinical practice is necessary to confirm its actual utility as a biomarker.

## 5 Conclusions

In summary, this study not only validated the correlation between CyPA levels and the risk of STEMI but also provided comprehensive evidence through various statistical analyses, highlighting the potential clinical utility of CyPA as a biomarker. Future research should further investigate the mechanisms through which CyPA operates in different cardiovascular diseases, particularly its specific role in patients with STEMI. This will aid in uncovering CyPA as a potential therapeutic target in cardiovascular diseases and provide a theoretical foundation for the development of novel treatment strategies. Moreover, the incorporation of other biomarkers may further enhance the accuracy of risk assessment for STEMI, offering more comprehensive decision support for clinical practices. Large-scale, multicenter clinical studies are needed to validate the effectiveness and reliability of CyPA as a biomarker, thereby establishing a solid foundation for its application in clinical practice.

## Data Availability

The raw data supporting the conclusions of this article will be made available by the authors, without undue reservation.
