# Supplementary material for: Correlation between serum cyclophilin A and acute ST-segment elevation myocardial infarction and its predictive value
Source: Front Cardiovasc Med. 2025 May 27;12:1566129. doi: 10.3389/fcvm.2025.1566129 (PMC12149216; doi:10.3389/fcvm.2025.1566129)
Supplement: Supplementary file 1 [file Table1.pdf]

Correlation between serum cyclophilin A and acute ST-segment elevation myocardial infarction and its predictive value

Yuqi Wang[1,2],Long Wang[2],Caoyang Fang[3,*],Zhenfei Chen[1,2,*]

[1]Graduate School, Bengbu Medical University, Longzi Lake District, Bengbu, Anhui 233030, China;

[2]Department of Cardiology,The Second People’s Hospital of Hefei, Hefei Hospital Affiliated to Anhui Medical University, Hefei, Anhui 230011, China;

[3]Department of Emergency,First Affiliated Hospital of University of Science and Technology of China,Anhui Provincial Hospital, Hefei, Anhui,230000, China;

***Corresponding Author:**

Caoyang Fang,Department of Emergency,First Affiliated Hospital of University of Science and Technology of China,Anhui Provincial Hospital, Hefei, Anhui,230000, China;E-mail:fcyahslyy@ustc.edu.cn

Zhenfei Chen,Department of Cardiology,The Second People’s Hospital of Hefei, Hefei Hospital Affiliated to Anhui Medical University, Hefei, Anhui 230011, China;E-mail:1601994492@qq.com

Supplementary Table 1.Univariate and multivariate logistic regression analysis

	Univariate Logistic Regression Analysis					Multivariate Logistic Regression Analysis				
	**β**	**S.E**	**Z**	***P***	**OR (95%CI)**	**β**	**S.E**	**Z**	***P***	**OR (95%CI)**
**Sex**										
Female					1.00 (Reference)					1.00 (Reference)
Male	1.45	0.21	6.95	<0.001	4.27 (2.84 ~ 6.43)	1.00	0.54	1.84	0.065	2.71 (0.94 ~ 7.79)
**Smoke**										
No					1.00 (Reference)					
Yes	0.25	0.18	1.41	0.158	1.28 (0.91 ~ 1.82)					
**Alcohol**										
No					1.00 (Reference)					1.00 (Reference)
Yes	-1.29	0.25	-5.21	<0.001	0.28 (0.17 ~ 0.45)	-4.25	0.87	-4.90	<0.001	0.01 (0.00 ~ 0.08)
**Hypertension**										
No					1.00 (Reference)					1.00 (Reference)
Yes	2.73	0.25	10.86	<0.001	15.37 (9.39 ~ 25.18)	5.16	0.85	6.10	<0.001	173.95 (33.18 ~ 912.04)
**Diabetes**										
No					1.00 (Reference)					1.00 (Reference)
Yes	-2.38	0.21	-11.58	<0.001	0.09 (0.06 ~ 0.14)	-4.32	0.74	-5.85	<0.001	0.01 (0.00 ~ 0.06)
**Age**	1.67	580.39	0.00	0.998	5.30 (0.00 ~ Inf)					
**BMI**	-0.04	0.02	-1.81	0.070	0.96 (0.91 ~ 1.00)					
**Neutrophils**	0.74	0.06	11.73	<0.001	2.10 (1.86 ~ 2.38)	0.80	0.13	6.03	<0.001	2.23 (1.72 ~ 2.89)
**Lymphocytes**	-0.11	0.11	-0.99	0.320	0.90 (0.72 ~ 1.11)					
**Monocyte**	5.14	0.59	8.73	<0.001	171.46 (54.05 ~	3.05	1.55	1.96	0.050	21.08 (1.01 ~ 441.56)

	Univariate Logistic Regression Analysis					Multivariate Logistic Regression Analysis				
	**β**	**S.E**	**Z**	***P***	**OR (95%CI)**	**β**	**S.E**	**Z**	***P***	**OR (95%CI)**
					543.93)					
**HB**	0.03	0.01	4.68	<0.001	1.03 (1.02 ~ 1.04)	0.04	0.02	1.79	0.073	1.04 (1.00 ~ 1.09)
**Platelets**	0.01	0.00	2.00	0.046	1.01 (1.01 ~ 1.01)	-0.00	0.00	-0.38	0.707	1.00 (0.99 ~ 1.00)
**Albumin**	-0.14	0.03	-4.78	<0.001	0.87 (0.82 ~ 0.92)	-0.14	0.07	-1.99	0.047	0.87 (0.76 ~ 0.99)
**Creatinine**	0.02	0.01	4.06	<0.001	1.02 (1.01 ~ 1.03)	0.00	0.02	0.05	0.962	1.00 (0.97 ~ 1.04)
**BUN**	0.08	0.05	1.73	0.083	1.08 (0.99 ~ 1.19)					
**eGFR**	0.01	0.01	1.09	0.276	1.01 (1.00 ~ 1.02)					
**UA**	0.01	0.00	2.40	0.017	1.01 (1.01 ~ 1.01)	-0.00	0.00	-0.43	0.667	1.00 (0.99 ~ 1.00)
**FPG**	0.26	0.05	4.95	<0.001	1.29 (1.17 ~ 1.43)	0.29	0.13	2.21	0.027	1.34 (1.03 ~ 1.74)
**TG**	0.15	0.09	1.70	0.090	1.17 (0.98 ~ 1.39)					
**TC**	0.41	0.08	4.84	<0.001	1.51 (1.28 ~ 1.78)	0.07	0.30	0.24	0.807	1.08 (0.59 ~ 1.95)
**LDL**	0.61	0.10	5.91	<0.001	1.85 (1.51 ~ 2.27)	0.85	0.34	2.51	0.012	2.33 (1.20 ~ 4.52)
**HDL**	-2.08	0.36	-5.71	<0.001	0.12 (0.06 ~ 0.26)	-2.82	1.15	-2.46	0.014	0.06 (0.01 ~ 0.57)
**HbA1c**	-0.05	0.05	-0.99	0.325	0.95 (0.86 ~ 1.05)					
**LVEF**	-0.12	0.02	-7.07	<0.001	0.89 (0.86 ~ 0.92)	-0.16	0.05	-3.20	0.001	0.85 (0.77 ~ 0.94)
**CyPA**	0.11	0.01	9.15	<0.001	1.11 (1.09 ~ 1.14)	0.14	0.03	4.75	<0.001	1.15 (1.09 ~ 1.23)

BMI:Body mass index,HB:Hemoglobin,BUN:Blood urea nitrogen,eGFR:Estimated glomerular filtration rate,UA:Uric acid,FPG:Fasting plasma glucose,TG:Triglyceride,TC:Total cholesterol,HDL:High density lipoprotein,LDL:Low density lipoprotein,HbA1c:Glycosylated hemoglobin,LVEF:Left ventricular ejection fraction,CyPA:Serum cyclophilin A
